# Fabrication of Sb^3+^ sensor based on 1,1′-(-(naphthalene-2,3-diylbis(azanylylidene))bis(methanylylidene))bis(naphthalen-2-ol)/nafion/glassy carbon electrode assembly by electrochemical approach†

**DOI:** 10.1039/c8ra01827h

**Published:** 2018-05-29

**Authors:** Mohammed M. Rahman, Tahir Ali Sheikh, Reda M. El-Shishtawy, Muhammad Nadeem Arshad, Fatimah A. M. Al-Zahrani, Abdullah M. Asiri

**Affiliations:** Chemistry Department, Faculty of Science, King Abdulaziz University Jeddah 21589 Saudi Arabia mmrahman@kau.edu.sa elshishtawy@hotmail.com aasiri2@kau.edu.sa; Center of Excellence for Advanced Materials Research (CEAMR), Faculty of Science, King Abdulaziz University Jeddah 21589 Saudi Arabia; Dyeing, Printing and Textile Auxiliaries Department, Textile Research Division, National Research Centre Dokki Cairo Egypt; Chemistry Department, Faculty of Science, King Khalid University Abha Saudi Arabia

## Abstract

A new Schiff base named 1,1′-(-(naphthalene-2,3-diylbis(azanylylidene))bis (methanylylidene))bis(naphthalen-2-ol) (NDNA) derived from 2,3-naphthalenediamine and 2-hydroxy-1-naphthaldehyde was synthesized by condensation reaction and then characterized by spectroscopic techniques for structure elucidation. In addition to spectroscopic techniques, the molecular structure of NDNA was clearly confirmed by single-crystal X-ray diffraction study. A thin film of NDNA was fabricated onto glassy carbon electrode (GCE) using 5.0% ethanolic nafion solution as a conducting binder in order to develop the cationic electrochemical sensor (NDNA/nafion/GCE) for the sensing of heavy-metal cations in aqueous systems by electrochemical technique. This newly designed sensor exhibited higher sensitivity and selectivity towards antimony (Sb^3+^) in the presence of other interfering heavy metal cations, as well as long-term stability. Fascinating analytical parameters such as limit of detection (LOD = 0.075 nM, SNR of 3), limit of quantification (LOQ = 0.25 nM) and sensitivity (12.658 × 10^−4^ μA μM^−1^ cm^−2^) were calculated from the calibration curve plot, which shows a linear dynamic range (LDR) of Sb^3+^ ion concentration from 0.1–10.0 mM. This work presents a new approach towards the development of sensitive, efficient as well as selective toxic cationic electrochemical sensors in the environmental and healthcare fields. Hence, this newly designed NDNA/nafion/GCE presents cost-effective and efficient outcomes and can be used as a practical substitute for the efficient detection and removal of Sb^3+^ ions from water samples.

## Abstract 2

A new Schiff base named 1,1′-(-(naphthalene-2,3-diylbis(azanylylidene))bis(methanylylidene))bis(naphthalen-2-ol) (NDNA) was synthesized by condensation reaction and then characterized by spectroscopic techniques for structure elucidation.

## Graphical abstract

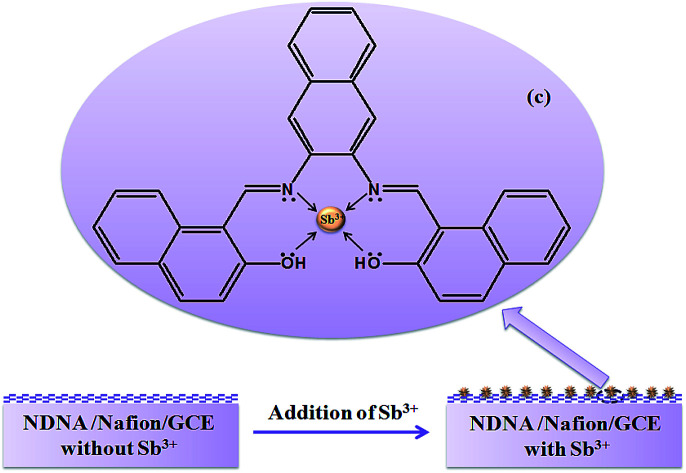

## 1. Introduction

Heavy-metal ions badly affect the aquatic environment, consequently damaging human health. The pollution by heavy metals occurs *via* many routes, such as industrial effluents, refineries, waste-treatment plants, groundwater and rainwater.^1^ Therefore, studies are growing toward making suitable devices for sensing and detecting such traces in the environment.^2–4^ In this interest, the Schiff base, which is the condensation product of aldehyde (or ketone) and amine-containing compounds,^5,6^ seems to be a viable ligand for sensing metal ions.^4,7,8^ Schiff bases have shown several biological activities such as antibacterial, antifungal, antimalarial, anti-inflammatory, and antipyretic.^9^ These properties are mainly due to the imine group present in Schiff bases, which could also be altered by varying the substituents in the molecules.^10–12^ Furthermore, several studies have explored the properties of Schiff bases as catalysts,^13–16^ fluorescent materials,^17–19^ electroluminescent materials,^20–22^*etc.* Since Schiff bases are known by their strong coordinative ability as a family of ligands, with the formation in most cases of 1 : 1 transition metal complexes,^23^ they have been used to develop various chemosensors.^24–26^ Chemosensors can be classified into three categories according to the nature of the signal emitted by the signaling subunit: (i) colorimetric sensors related to change in electronic properties in the form of intra/intermolecular charge transfer (ICT),^27–29^ (ii) fluorogenic sensors related to photoinduced electron transfer (PET),^27,30^ excited-state intramolecular proton transfer (ESIPT),^31^ fluorescence resonance energy transfer (FRET),^32,33^ bond energy transfer (TBET),^34^ excimer–exciplex formation,^35^*etc.*, and (iii) electrochemical sensors related to measurement of changes in redox potential or electrical responses. Because of the great worth and commercial applications of the Schiff bases, the intention of this study is to design an ion-selective cationic electrochemical sensor based on a newly synthesized, non-reported Schiff base as tetradentate ligand in order to probe heavy metal ions in an aqueous system by electrochemical approach. For this reason, 2,3-naphthalenediamine and 2-hydroxy-1-naphthaldehyde were selected as precursors to synthesize the novel Schiff base, named as NDNA. It was observed that the newly designed cationic electrochemical sensor based on the above Schiff base is selective for Sb^3+^ in the presence of other interfering heavy metal ions in aqueous system.

The toxicity of antimony on health includes keratitis, dermatitis, gastritis, and conjunctivitis, and long-term exposure can cause the development of cardiac problems and lung cancer.^36,37^ Aquatic environments are polluted by Sb through rock weathering, soil runoff and anthropologic activities.^38,39^ The toxicity of antimony strongly depends on its oxidation state, as (Sb^3+^) is about ten times more toxic than (Sb^4+^).^40,41^ The maximum permissible concentrations in drinking water as recommended by the U.S. Environmental Protection Agency (EPA) and World Health Organization (WHO) are 6 and 20 mg L^−1^, respectively.^42,43^ Even though concentrations as low as 1 mg L^−1^ Sb can be found in a non-polluted water sample,^44^ high concentrations of Sb can reach 100 mg L^−1^ in nearby anthropogenic sources.^45^ Therefore, the detection and monitoring of Sb have been compulsory. Various analytical approaches such as UV-Vis spectrophotometer,^46,47^ capillary electrophoresis,^48^ spectrofluorimetry,^49^ atomic absorption spectrometry,^50–54^ laser-induced fluorescence,^55^ inductively coupled plasma-optical emission spectrometry (ICP-OES),^42,56^ high-performance liquid chromatography coupled with hydride generation-atomic fluorescence spectrometry (HPLC-HG-AFS),^57,58^*etc.* have been reported for the determination of antimony (Sb^3+^) in addition to the various electrochemical methods.^59,60^ Voltammetric well as potentiometric methods based on modified mercury electrodes as working electrodes have been reported for the detection of antimony.^61,62^ Many of the reported methods are not very effective with respect to their detection limit. If some are effective, then they are very complicated and not very cost-effective. So, it has become our need to develop a cost-effective, reliable and efficient method for the effective determination of Sb^3+^. In this research, current–voltage (*I*–*V*) technique, an electrochemical approach, was applied for the effective determination of Sb^3+^ by using a newly designed, selective and efficient electrochemical sensor (NDNA/nafion/GCE) in a lab-made electrochemical cell which accommodates aqueous solution. Glassy carbon electrode (GCE) was used as a working electrode/sensor for the trace determination of Sb^3+^. It was modified by the newly synthesized Schiff base (NDNA) coated onto its flat surface with 5% ethanolic nafion as a conducting binder. The modified electrode shows very sensitive and excellent transduction in phosphate buffer solution (PBS, 0.1 M at 7.0 pH) at the interface of the liquid and electrode surface. Per our knowledge, this is the first cationic sensing application based on the lab-made tetradentate NDNA Schiff base as chelating agent for the highly sensitive, selective and rapid determination of antimony(iii) in aqueous solution, qualitatively and quantitatively, using *I*–*V* method with a short response time.

## 2. Experimental

### 2.1. Materials and methods

All solvents and reagents were purchased from Sigma-Aldrich Company and used as received. ^1^H and ^13^C NMR spectra were recorded in DMSO-d6 solutions on a Bruker Avance 850 MHz spectrometer. Infrared spectra were performed on a PerkinElmer spectrum 100 FTIR spectrometer. FT-IR spectra were recorded as neat on a Thermo Scientific NICOLET iS50 FT-IR spectrometer (Thermo Scientific, Madison, WI, USA). Melting points were determined in open capillary tubes in a Stuart Scientific melting point apparatus (SMP3) and are uncorrected.

### 2.2. X-ray crystallography of compound I (NDNA)

To observe the geometry of molecules and their interactions in the unit cell, we have crystalized the compound I (NDNA). Good-looking crystals were taken to the microscope for the final selection of sample for mounting on the diffractometer. The selected crystal was fixed over the tip of a thin glass fiber adsorbed in wax on a copper rod with a magnetic base. This holder was mounted on an Agilent SuperNova (dual source) Agilent Technologies Diffractometer, equipped with graphite-monochromatic Cu/Mo Kα radiation source for data collection. The data collection was accomplished using the CrysAlisPro software^63^ at 296 K under Cu Kα radiation. The structure solution was performed and refined by full-matrix least-squares methods on *F*^2^ using SHELXL-97,^64^ in-built with WinGX.^65^ All non-hydrogen atoms were refined anisotropically by full-matrix least-squares methods.^64^ Figures were drawn using PLATON^66^ and ORTEP-3.^67^

All the aromatic hydrogen atoms were positioned geometrically and treated as riding atoms with C–H = 0.93 Å and *U*_iso_ (H) = 1.2 *U*_eq_ (C) carbon atoms. The N–H = 0.99(6) Å and O–H = 0.93(6) Å; the hydrogen atoms were located with the difference Fourier map and refined with *U*_iso_ (H) = 1.2 *U*_eq_ (N) and *U*_iso_ (H) = 1.5 *U*_eq_ (O), respectively. The CIF of the NDNA has been submitted to the Cambridge Crystallographic Data Centre (CCDC).

### 2.3. Synthesis of NDNA

A solution of 2,3-naphthalenediamine (1.58 g, 10 mmol) and 2-hydroxy-1-naphthaldehyde (3.44 g, 20 mmol) in 200 mL of 50/50 v/v ethanol/methanol (absolute) was stirred for 24 h at room temperature and then refluxed for 8 h, then left to cool. The orange precipitate was filtered and washed several times with ethanol to yield 4.15 g product (90% yield), re-crystallized from chloroform/methanol to obtain the single crystal for further analysis. Mp: 324–326 °C. ^1^H NMR (DMSO-d6, 850 MHz, *δ* = ppm): 7.09 (2H, d, *J* = 8.5 Hz, Ar–H), 7.40 (2H, t, *J* = 6.8 Hz, Ar–H), 7.57 (2H, m, Ar–H), 7.60 (2H, td, *J* = 7.22, 0.85 Hz, Ar–H), 7.85 (2H, d, *J* = 7.65 Hz, Ar–H), 7.99 (2H, d, *J* = 9.35 Hz, Ar–H), 8.10 (2H, m, Ar–H), 8.31 (2H, s, Ar–H), 8.61 (2H, d, *J* = 8.5 Hz, H–Ar), 9.83 (2H, s, N=CH), 15.08 (2H, s, OH); ^13^C NMR (DMSO-d6, 213 MHz, *δ* = ppm): 19.01, 109.89, 117.17, 121.16, 122.01, 124.22, 126.81, 127.41, 128.15, 128.73, 129.54, 132.59, 133.50, 137.47, 138.78, 157.89, 158.89, 169.36; FT-IR (ATR, cm^−1^): 3675, 3054, 1618, 1607, 1593, 1568, 1542, 1490, 1350, 1307, 1162, 863, 739.

### 2.4. Fabrication of electrochemical sensor

**Scheme 1:**
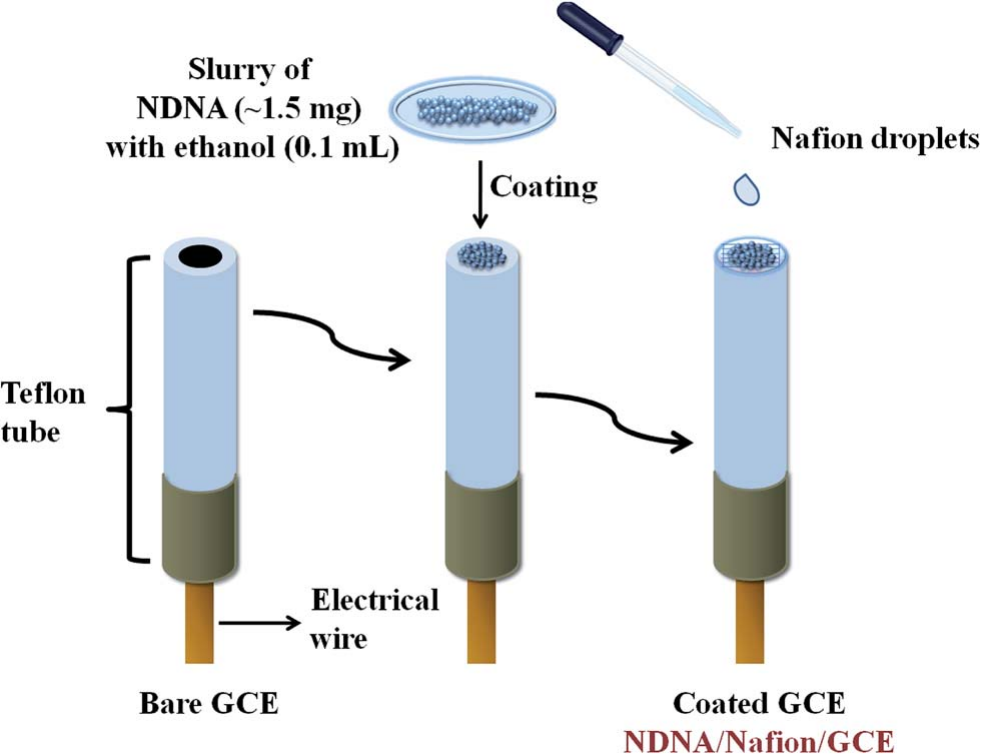
Fabrication of glassy carbon electrode modified by NDNA with the conducting binder nafion.

A very easy and effortless method was applied to fabricate the GCE with newly synthesized non-reported Schiff base as chelating agent. A very small amount (approximately 1.5 mg) of NDNA was mixed with 0.1 mL of ethanol in order to make the slurry, which was then applied onto the flat surface of the GCE, with one drop of 5% ethanolic nafion as an adhesive conducting binder, Scheme 1. Herein, nafion was used as an adhesive conducting polymer in order to adhere NDNA to the flat surface of GCE and allow the conduction of electrons between NDNA and GCE against the potential (*V*) applied in the system during the detection of toxic cations. After coating, it was dried at room temperature for 45 minutes to obtain the completely dried, evenly coated and stable NDNA/nafion/GCE as an efficient and selective cationic electrochemical sensor for Sb^3+^. A Pt wire was also used as counter electrode in addition to the newly designed NDNA/nafion/GCE as working electrode to measure the *I*–*V* response. In this way, a lab-made electrochemical cell was formed which accommodates the two electrodes (working and counter electrodes) to dip into phosphate buffer solution (PBS) of pH = 7.0, in a 15 mL beaker. The volume of PBS was kept constant at 10 mL in this lab-made electrochemical cell throughout the study. The PBS was prepared by mixing 39 mL of 0.2 M Na_2_HPO_4_ and 61 mL of 0.2 M NaH_2_PO_4_ in a 200 mL measuring cylinder, diluting to the mark by deionized (DI) water. The stock solution of Sb^3+^ was used to make the different concentrations of Sb^3+^ (full concentration range: 0.1 nM to 0.1 M) in DI water for use as our target analyte. Linear dynamic range (LDR), regression coefficient *r*^2^, sensitivity, limit of detection (LOD) at S/3N, and limit of quantification (LOQ) for Sb^3+^ was calculated from the slope of the calibration curve (from current *versus* concentration plot). Keithley electrometer was used as a constant voltage source for *I*–*V* measurement in a simple two-electrode system.

## 3. Results and discussion

### 3.1. Synthesis

**Scheme 2:**
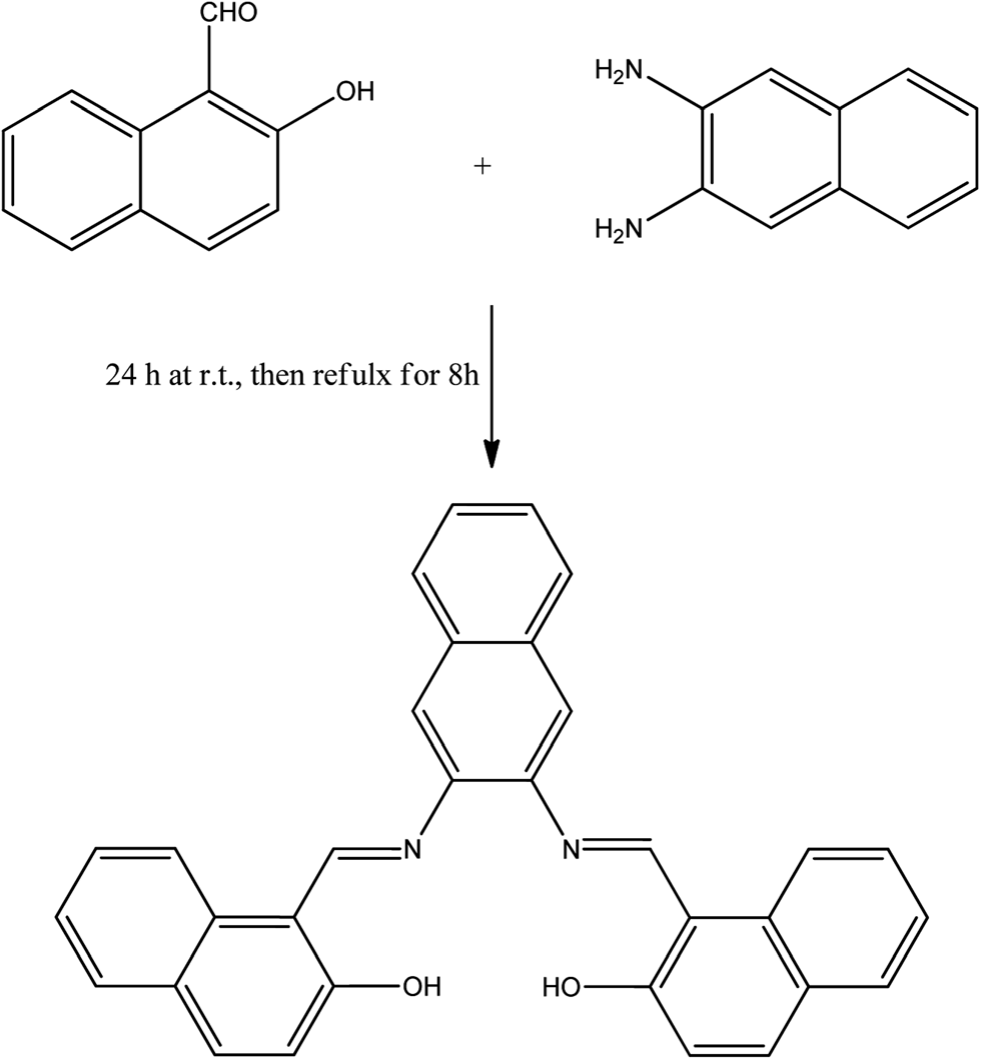
Synthesis of proposed NDNA compound.

**Scheme 3:**
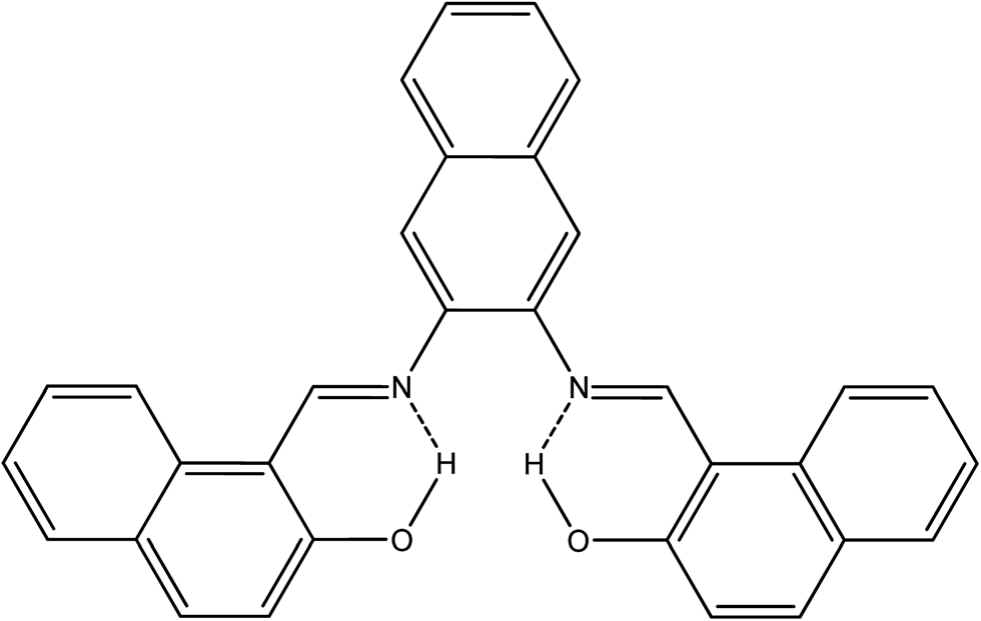
Expected intramolecular hydrogen bonds in NDNA.

The idea behind the present work is to have a bifunctional fused aromatic Schiff base that would be suitable for making a stable film on an electrochemical device. For this purpose, NDNA Schiff base was easily obtained (Scheme 2) in excellent yield by the condensation reaction of 2-hydroxynaphthaldehyde and 2,3-naphthalenediamine. ATR-FTIR, ^1^H NMR, ^13^C and single-crystal X-ray spectroscopy measurements were made to confirm the chemical structure of NDNA. The FTIR spectra show several characteristic peaks, for the azomethine group (CH=N) at 1618 and aromatic rings (C=C) at 1607, 1593, 1568, and 1542 cm^−1^. Additionally, C–H aromatic stretching vibration is clearly observed at 3054 cm^−1^. Other bands appear at 1490, 1350, and 1307 cm^−1^, attributed to C–H bending vibration, C–N stretching vibration and C–O stretching vibration, respectively. An *ortho*-di-substituted aromatic band appears at 739 cm^−1^. The phenolic O–H stretching vibration appears weak, with a broad peak centered at 3675 cm^−1^. This behavior might be due to intramolecular hydrogen bonding with the azomethine nitrogen atom. The proton NMR data show the imine and OH protons as singlet, far downfield at 9.83 and 15.08 ppm, respectively. This downfield effect is attributed to the formation of intramolecular hydrogen bonds, which results in further de-shielding of the OH group protons. Also, the creation of a six-membered ring as a result of intramolecular hydrogen bonds (see Scheme 3) may further impact the OH proton by the magnetic anisotropic effect. Previous studies indicated that intramolecular hydrogen bonds have a very significant effect on phenolic protons, with chemical shifts in the range of 4.5 to 19 ppm.^22^ Good correlation between doublet and triplet protons with their corresponding coupling constants are clearly indicative of the neighboring aromatic protons as presented in the experimental section. Additionally, ^13^C NMR recorded all carbons present in the NDNA, with a special attention to the characteristic peak of aromatic azomethine carbon at 169.36 ppm.

### 3.2. Single-crystal X-ray description of synthesized compound

**Fig. 1:**
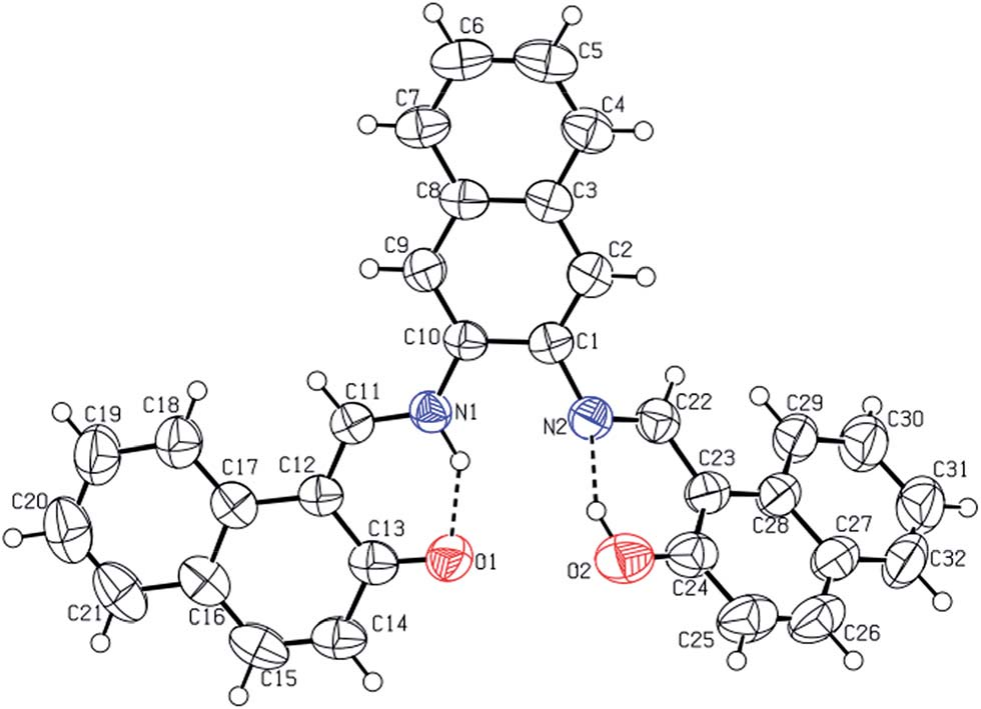
Labelled ORTEP diagram of molecule I (17082), where thermal ellipsoids were drawn at 50% probability level.

**Fig. 2:**
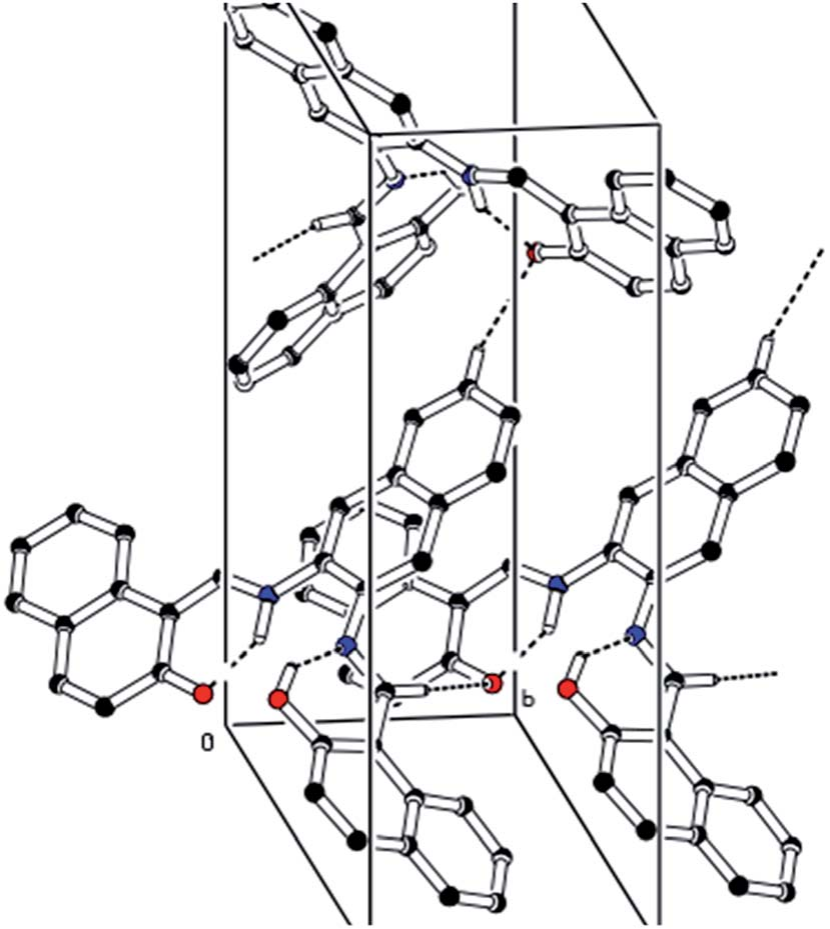
A unit cell view showing the inter- and intra-molecular hydrogen bonding using dashed lines.

The CCDC number (1812136) obtained for the NDNA is mentioned in Table 1. There are three naphthalene rings (C1–C10), (C12–C21) and (C23–C32) in the molecule (Fig. 1). The root mean square (r.m.s.) deviations for the fitted atoms of these naphthalene ring systems are 0.0086 Å, 0.0222 Å and 0.0363 Å. Bond angles and bond lengths are shown in Tables S1 and S2.† Two of the naphthalene rings are almost planar with respect to each other, as the dihedral angle between the (C1–C10) and (C12–C21) is 4.060 (4)°, while the dihedral angle between the naphthalene rings (C1–C10) and (C23–C32) is 60.694 (8)°. The molecule exhibits inter- and intra-molecular hydrogen bonding interactions. There are N–H⋯O and O–H⋯N type intramolecular interactions from the six-membered ring motifs shown in Fig. 1.^68^ The ring motif generated through the atoms (O1/C13/C12/C11/N1/H1n) have the r.m.s. deviation of 0.0301 Å, and its dihedral angle with its fused naphthalene ring is 3.298 (9)°. The second ring motif generated through the atoms (N2/C22/C23/C24/O2/H1o) has almost the same r.m.s. deviation, *i.e.* 0.0325 Å, and is twisted at a dihedral angle of 7.727 (1)° with respect to the naphthalene ring (C23–C32). The intermolecular hydrogen bonding interactions observed in the crystal structure of compound I are weak, but they connect the molecules to generate the intricate network. Atom C(6) in the molecule occupied at (*x*, *y*, *z*) takes part in hydrogen bonding and acts as donor atom *via* H(6) to atom O(1) at (1 − *X*, 1 − *Y*, 1/2 + *Z*). This interaction connects the molecules along the *c*-axis to form infinite long chains (Fig. 2 and S1,†Table 2). On the other hand, the atom C(22) at (*x*, *y*, *z*) acts as donor *via* H(6) to atom O(1) at (*X*, 1 + *Y*, *Z*) and connects the molecules along the *b*-axis (Fig. 2 and S1,†Table 2). Both interactions produce the network along the *bc* plane.

**Table 1:** Crystal data and structure refinement for I

CCDC code	1812136
Empirical formula	C_32_H_22_N_2_O_2_
Formula weight	566.51
Temperature/K	296(2)
Crystal system	Orthorhombic
Space group	*Pca*2_1_
*a*/Å	20.7011(17)
*b* Å	6.2155(4)
*c*/Å	18.0683(13)
α/°	90
β/°	90
γ/°	90
Volume/Å^3^	2324.8(3)
*Z*	4
*ρ*_calc_ mg mm^−3^	1.333
*μ*/mm^−1^	0.084
*F*(000)	976.0
Crystal size/mm^3^	0.29 × 0.21 × 0.14
2*θ* range for data collection	5.986 to 58.31°
Index ranges	−27 ≤ *h* ≤ 26, −8 ≤ *k* ≤ 5, −24 ≤ *l* ≤ 24
Reflections collected	13 650
Independent reflections	5313[*R*_(int)_ = 0.0505]
Data/restraints/parameters	5313/1/332
Goodness-of-fit on *F*^2^	1.042
Final *R* indexes [*I* ≥ 2*σ*(*I*)]	*R*_1_ = 0.0543, w*R*_2_ = 0.1103
Final *R* indexes [all data]	*R*_1_ = 0.1126, w*R*_2_ = 0.1369
Largest diff. peak/hole/e Å^−3^	0.14/−0.14

**Table 2:** Hydrogen bonds for 17082

D	H	A	*d*(D–H)/Å	*d*(H–A)/Å	*d*(D–A)/Å	D–H–A/°
C6	H6	O1^a^	0.93	2.55	3.278(5)	135.0
C22	H22	O1^b^	0.93	2.50	3.363(5)	155.4
N1	H1N	O1	0.99(6)	1.68(6)	2.547(4)	144(4)
O2	H1O	N2	0.93(6)	1.76(6)	2.580(5)	146(6)

a 1 − *X*, 1 − *Y*,1/2 + *Z*.

b +*X*, 1 + *Y*, +*Z*.

### 3.3. Application: detection of Sb^3+^ ions with NDNA/nafion/GCE probe

The impressive application of NDNA is the electrochemical sensing of heavy metal cations in aqueous solution by using *I*–*V* approach. The fabrication of GCE with NDNA as chelating agent has been discussed in the experimental section. The creation of the NDNA/nafion/GCE as an efficient and selective cationic electrochemical sensor in a two-electrode system (working and counter electrodes) is a new report as per our knowledge, and no other reports are found in the literature. Moreover, the sensor was found to be very selective and sensitive for Sb^3+^ in the presence of other interfering heavy metal cations. The notable change in current response of the newly designed NDNA/nafion/GCE against applied potential was observed in the presence of our target analyte in aqueous solution upon being adsorbed on the surface of the working electrode. After the fruitful result of a change in the current response of the modified GCE, analytical parameters such as LDR, LOD, LOQ, *etc.* were also studied in order to optimize the newly designed NDNA/nafion/GCE as an efficient, sensitive and as well as selective cationic electrochemical sensor for Sb^3+^.

**Fig. 3:**
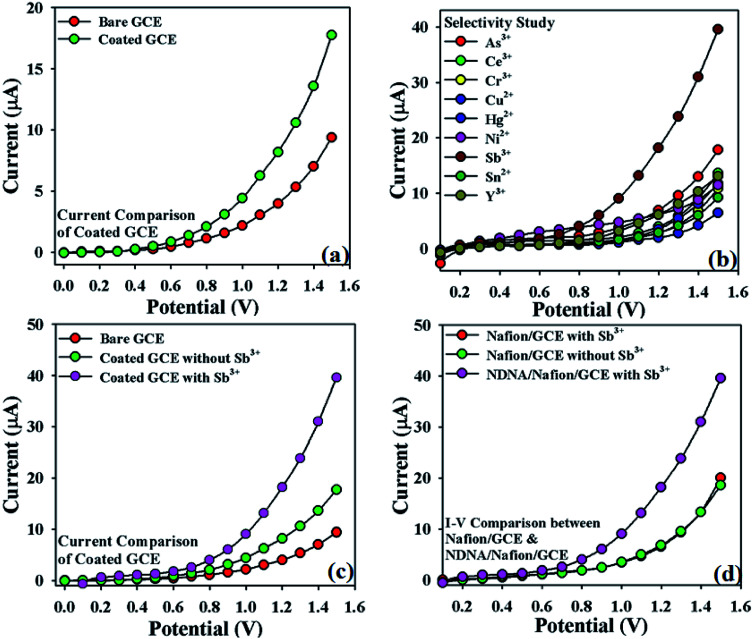
Selectivity study: (a) *I*–*V* response of bare and NDNA-coated GCE; (b) *I*–*V* response with various analytes (showing affinity with Sb^3+^), concentration of each analyte was taken at 0.1 μM, 25 μL; (c) comparison of *I*–*V* response of bare and NDNA/nafion/GCE with and without the target analyte Sb^3+^ at 0.1 μM, 25 μL; (d) comparison of *I*–*V* response of nafion/GCE and NDNA/nafion/GCE with and without the target analyte.

Initially, the change in current response with this newly designed NDNA/nafion/GCE against the applied potential was observed and compared with the bare GCE in the absence of target analyte. It was noticed that the fabricated GCE has a high current response as compared to the bare GCE, which reflects the high electron communication feature between the active site of NDNA and GCE (Fig. 3a). Subsequently, a selectivity study was carried out in the presence of various heavy metal cations such as Sb^3+^, As^3+^, Ce^3+^, Cr^3+^, Cu^2+^, Hg^2+^, Ni^2+^, Sn^2+^ and Y^3+^, and it was found to be very selective only for Sb^3+^ (Fig. 3b). For selectivity study, 25 μL of 0.1 μM concentration of toxic cations was used. Moreover, the current response in the presence and absence of Sb^3+^ was also observed in order to confirm the fabricated GCE's affinity with Sb^3+^, and it was found that it shows very good response in the presence of Sb^3+^ (Fig. 3c). It also reflects the very good adsorption and absorption capability of the Sb^3+^analytes onto the coated surface of the GCE. Similarly, the *I*–*V* response of nafion/GCE in the presence and absence of Sb^3+^ analytes was also observed in order to confirm that nafion, as conducting binder, also has no significant effect without NDNA for the sensing of Sb^3+^ in the system. It was noticed that the response of nafion/GCE without NDNA was almost same and has no significant effect in the presence and absence of Sb^3+^ (Fig. 3d). PBS solutions of different pH values were also investigated in order to optimize the operational condition of the newly designed GCE (Fig. S1 presented in ESI†). It was found that this newly designed GCE gives good response at pH 7.0 and 1.0 second as delay time in the presence of Sb^3+^.

**Fig. 4:**
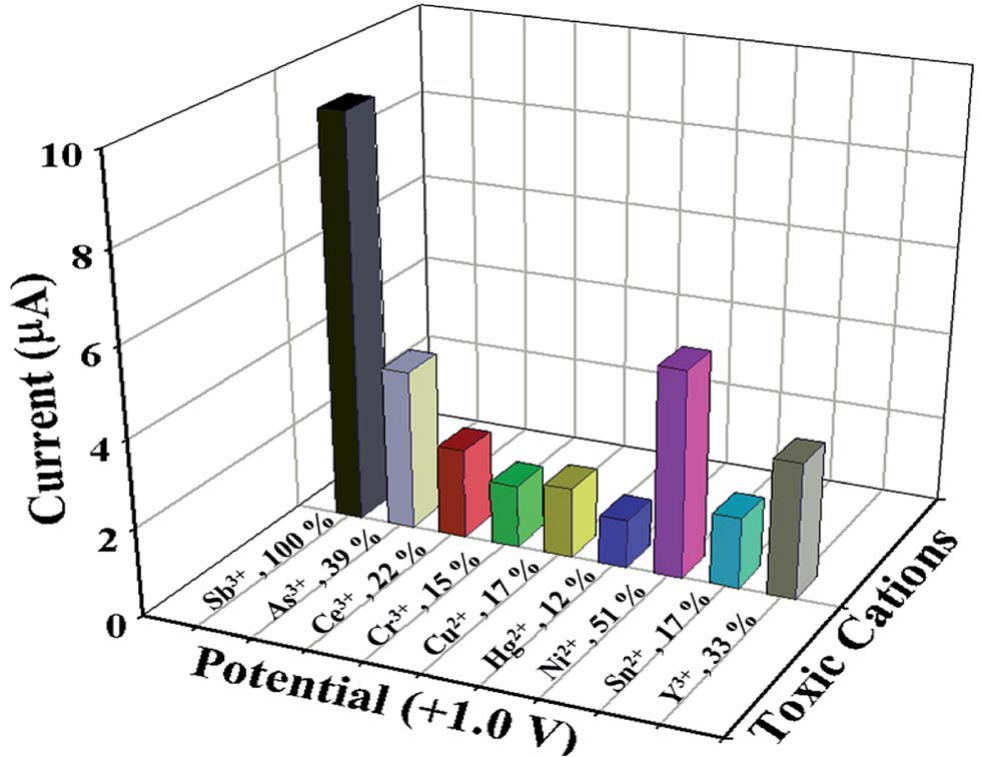
Interference study: comparison of *I*–*V* responses to interfering heavy metal cations at +1.0 V. Analyte concentrations were taken as 0.1 μM, delay time 1.0 s.

Similarly, statistical approach was also applied to study the interference effect of other heavy metal cations (As^3+^, Ce^3+^, Cr^3+^, Cu^2+^, Hg^2+^, Ni^2+^, Sn^2+^ and Y^3+^) on NDNA/nafion/GCE in the presence of Sb^3+^ analytes, in the form of *I*–*V* response at +1.0 V at normal condition, and results are presented in Fig. 4 and Table 3. The concentration and volume of all cations were kept constant, and the amounts were taken as 25 μL of 0.1 μM cation in PBS (7.0 pH). From the interference study, it was also concluded that NDNA/nafion/GCE is very selective toward Sb^3+^ detection. It does not exhibit any major change in current response towards the other interfering heavy metal cations compared to Sb^3+^. For the Sb^3+^, it has given a 3-fold current response compared to Ni^2+^, which gave the second highest current response.

**Table 3:** Interference effect of various cations with NDNA/nafion/GCE^a^

Metal ions	Observed current (μA)				Interference effect (%)	SD (*n* = 3)	RSD (%) (*n* = 3)
	*R*_1_	*R*_2_	*R*_3_	Average			
Sb^3+^	9.0214	9.2104	9.6461	9.2926	100	0.320	3.44
As^3+^	3.7026	3.1622	4.2134	3.6927	39	0.525	14.23
Ce^3+^	2.0452	2.5146	1.6454	2.0684	22	0.435	21.03
Cr^3+^	1.3736	1.5109	1.4631	1.4492	15	0.069	4.80
Cu^2+^	1.5437	1.3383	1.9694	1.6171	17	0.321	19.90
Hg^2+^	1.0972	0.9170	1.3465	1.1202	12	0.215	19.25
Ni^2+^	4.7218	5.0739	4.4944	4.7633	51	0.291	6.12
Sn^2+^	1.5799	1.4827	1.8366	1.6330	17	0.182	11.19
Y^3+^	3.1301	2.4540	3.6794	3.1301	33	0.613	19.87

a Interference effect of Sb^3+^ is considered to be 100%; *R* = reading; SD = standard deviation; and RSD = relative standard deviation; *n* = number of readings.

**Scheme 4:**
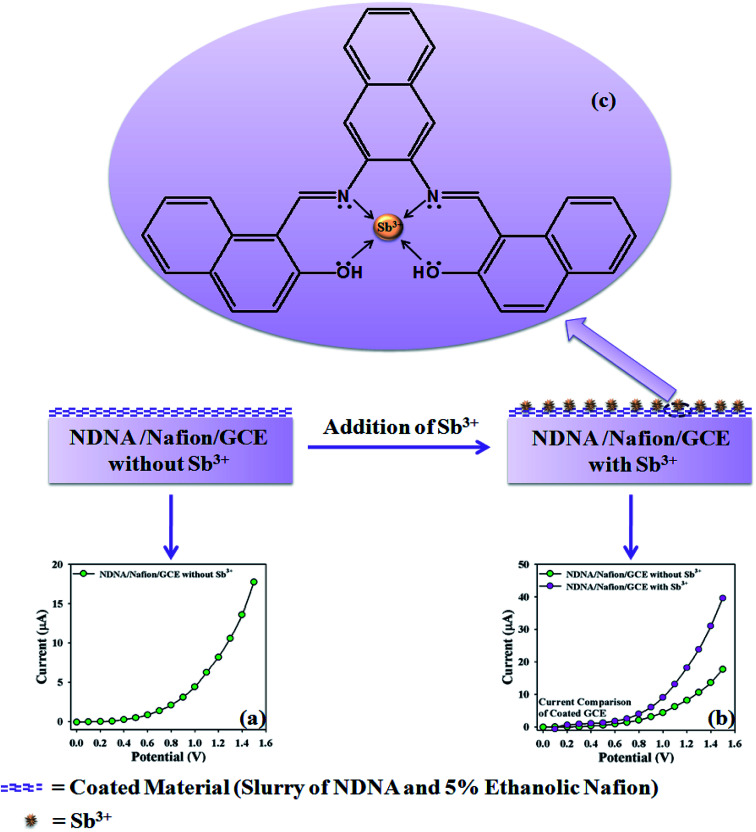
Proposed mechanism of the probable interaction of Sb^3+^ with NDNA/nafion/GCE, (a) *I*–*V* response without Sb^3+^, (b) comparison of *I*–*V* response with and without Sb^3+^, (c) probable π–π* interaction between Sb^3+^ ions and NDNA.

**Fig. 5:**
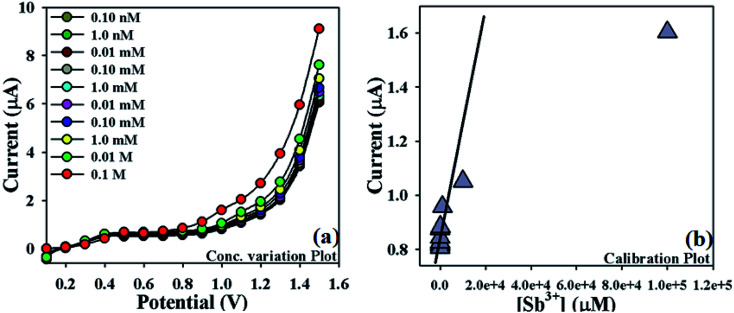
Optimization of newly designed Sb^3+^ sensor: (a) concentration variation plot of Sb^3+^ (0.1 M to 0.1 nM), (b) calibration plot (at +1.0 V) of NDNA/nafion/GCE.

The proposed mechanism of Sb^3+^ detection by using NDNA/nafion/GCE is shown in Scheme 4, with *I*–*V* graphical responses. *I*–*V* response of the newly designed NDNA/nafion/GCE in the presence of Sb^3+^ ions is functional in PBS at normal condition, and good response is observed. The current response of NDNA/nafion/GCE in the absence of Sb^3+^ is also presented in Scheme 4a in order to compare the current response in the presence of Sb^3+^ (Scheme 4b). When we add a small amount of Sb^3+^ into the system, there is a small increase in the current response of NDNA/nafion/GCE because a smaller surface of coated GCE is covered by the Sb^3+^ analytes (π–π* interaction). In this way, the surface reaction proceeds continuously but slowly and surely. Similarly, current response increases progressively with gradual increase of the concentration of Sb^3+^ analytes in the system (π–π* interaction). Subsequently, we can get a better idea about the increases in current response from the concentration variation plot (Fig. 5a). The surface reaction does not stop at this stage but increases progressively as a function of the concentration of our target analytes. Thus, we observe a greater current response of the NDNA/nafion/GCE-film as more surface is covered by our target analytes, along with increasing π–π* interaction of the functional groups of NDNA and Sb^3+^ (Scheme 4b). The π–π* interaction could be approached as inter-molecular and intra-molecular interactions of the functional compound. Similarly, the same phenomena have also been reported for the detection of toxic chemicals in the literature.^2,69^ Finally, Sb^3+^analytes on the NDNA/nafion/GCE surface reach the ideal saturated stage, which results in a regular increment of current response (Scheme 3c).

In order to optimize the NDNA/nafion/GCE as an efficient cationic electrochemical sensor for Sb^3+^, current responses at different concentrations of Sb^3+^ analytes in aqueous system against the applied potential were also observed. It was noticed that current increases as a function of Sb^3+^ concentration from lower to higher values (0.1 nM to 0.1 M) at normal condition (Fig. 5a). Analytical parameters such as linear dynamic range (LDR), regression coefficient (*r*^2^), limit of detection (LOD) and limit of quantification (LOQ) as well as sensitivity were found by plotting the calibration curve at the potential of +1.0 V from various concentrations of Sb^3+^ analytes (Fig. 5b). From the plot, the linear dynamic range (LDR), regression coefficient (*r*^2^), sensitivity, and limits of detection (LOD) and quantification (LOQ) were calculated as 0.1 nM to 10.0 mM, (*r*^2^) = 0.9710, 12.658 × 10^−4^ μA μM^−1^ cm^−2^, 0.075 nM and 0.25 nM, respectively.

**Fig. 6:**
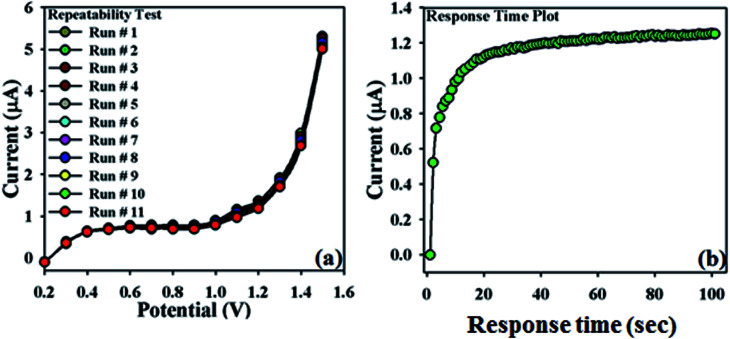
Repeatability and response time: (a) repeatability test (1 to 11 runs) with 25 μL of 0.1 μM Sb^3+^, (b) response time of 10 to 13 seconds to reach the saturated steady state.

In order to validate this newly designed NDNA/nafion/GCE, the repeatability test was carried out at the concentration of 0.1 μM with a sequence of nine to ten successive measurements (Fig. 6a). No significant changes were observed in current responses in addition to the absence of electrode poisoning and erosion during the detection of Sb^3+^ at normal condition. *I*–*V* response remained almost the same as the initial response after washing for each experiment. On the other hand, Fig. 6b shows the plot of response time of the newly designed NDNA/nafion/GCE recorded as current (μA) *vs.* time (sec) (*I*–*T*) measurement. It took 10 to 13 seconds to reach the saturated steady-state current. This plot indicates that the newly designed NDNA/nafion/GCE is very efficient for the detection of Sb^3+^ analytes even at very low concentration and within a short response time.

The sensitivity of the newly designed NDNA/nafion/GCE is due to its outstanding absorption (assembly of NDNA-modified GCE) and adsorption (surface of NDNA/nafion/GCE) properties. In addition, high catalytic decomposition characteristics and high electron communication features in the active sites of NDNA and GCE make it sensitive. The sensitivity and detection limit of this cationic sensor are in good according with the previously reported methods for the detection of Sb^3+^. The NDNA/nafion/GCE system introduces a very simple and reliable method to detect toxic chemicals, and it also reveals a significant access to a large group of chemicals for wide-ranging applications in environmental and healthcare fields. This study is the initial report for the sensitive and selective detection of Sb^3+^ by *I*–*V* technique based on the NDNA/nafion/GCE as compared to other previously reported methods. Table 4 shows the evaluation of the proposed *I*–*V* technique for the detection of Sb^3+^ using NDNA/nafion/GCE compared with other previously reported analytical techniques. It indicates that this outstanding proposed method, with our designed NDNA/nafion/GCE as a sensor for probing the Sb^3+^, is more sensitive and efficient than previously reported methods.

**Table 4:** Comparison of proposed electrochemical method with different previously reported analytical methods for the detection of Sb^3+^^a^

Methods	Material	Sensitivity	LDR	LOD	LOQ	Ref.
UV-Vis spectroscopy/PLS	Pyrogallol as complexing agent	—	1.0 × 10^−6^ to 1.0 × 10^−4^ M	39 800 nM	—	González *et al.*^46^
UV-Vis spectroscopy/CPE	DCHNAQ-CTAB	—	1.6 × 10^−9^ to 1.6 × 10^−7^ M	0.45 nM	1.51 nM	El-Sharjawy *et al.*^47^
Spectrofluorimetry	2ClRAAP	—	1.6 × 10^−9^ to 8.2 × 10^−8^ M	1.35 nM	—	Yu *et al.*^49^
SPE-ETAAS	APDC/carbon nanotubes	—	4.1 × 10^−10^ to 3.28 × 10^−8^ M	0.41 nM	—	López-García *et al.*^51^
ICP-OES	Immobilized l-proline	—	0.0–4.1 × 10^−7^ M	0.74 nM	—	Menegário *et al.*^56^
HPLC-HG-AFS	Anion exchange column PRP-X100	—	4.1 × 10^−9^ to 1.6 × 10^−6^ M	0.574 nM	—	De Gregori *et al.*^58^
Adsorptive cathodic stripping voltammetry	Morin/SMDE	—	1.0 × 10^−9^ to 3.0 × 10^−7^ M	0.7 nM	—	Zhou *et al.*^59^
Anodic stripping voltammetry	Pot. ferrocyanide/SPGE	—	8.2 × 10^−9^ to 7.47 × 10^−6^ M	4.76 nM	—	V. Kolliopoulos *et al.*^60^
Square wave voltammetry	HMDE	—	4.1 × 10^−9^ to 6.5 × 10^−8^ M	1.0 nM	—	Locatelli *et al.*^61^
Potentiometric	*p*-DMPF/HMDE	—	8.2 × 10^−8^ to 4.1 × 10^−7^ M	0.40 nM	—	Zhang *et al.*^62^
***I*–*V* method NDNA/nafion/GCE**	**NDNA/nafion/GCE**	**12.658 × 10** ^**−4**^ **μA μM** ^**−1**^ **cm** ^**−2**^	**1.0 × 10** ^**−10**^ **to 0.01 M**	**0.075 nM**	**0.25 nM**	**This work**

a PLS = partial least squares; CPE = cloud point extraction; DCHNAQ-CTAB = 3-dichloro-6-(3-carboxy-2-hydroxy-1-naphthylazo)quinoxaline-cetyltrimethylammonium bromide; SPE-ETAAS = solid-phase extraction-electrothermal atomic absorption spectrometry; APDC = ammonium pyrrolidinedithiocarbamate; ICP-OES = inductively coupled plasma optical emission spectrometry; 2ClRAAP = 3-*o*-chlorophenyl-5-(2-arsenoxylphenylazo) rhodanine; HPLC-HG-AFS = high-performance liquid chromatography-hydride generation-atomic fluorescence spectrometry detection; SMDE = static mercury drop electrode; SPGE = screen printed graphite electrode; *p*-DMPF = *p*-dimethyl-aminophenyl-fluorone; HMDE = hanging mercury drop electrode; NDNA = 1′-(-(naphthalene-2,3-diylbis(azanylylidene))bis(methanylylidene))bis(naphthalen-2-ol); GCE = glassy carbon electrode.

### 3.4. Real sample analysis

Real sample analysis was carried out in order to validate this proposed *I*–*V* method by using NDNA/nafion/GCE through the standard addition method.^70^ Industrial effluents, a baby feeding bottle, a PVC food packing bag, and a mineral water bottle were used for this purpose. A fixed amount of each sample (25.0 μL) was analyzed in PBS (0.1 M, pH = 7.0). The results (Table 5) obtained by this method were in good accord with the proposed *I*–*V* technique, which indicates that this technique is also reliable, satisfactory and stable for analyzing real samples with the newly designed NDNA/nafion/GCE as an efficient and selective Sb^3+^ cationic electrochemical sensor.

**Table 5:** Real sample analysis of Sb^3+^ in various environmental samples^a^

Real samples	Amount of 3-CP added	No. of readings	Measured response in (μA)	% recovery	Mean (% recovery)	SD	RSD	SEM
Sb^3+^	0.1 μM, 25 μL	—	21.073	100	—	—	—	—
Industrial effluent	0.1 μM, 25 μL	*R*_1_	16.262	77.1	73.0	3.857	5.27	2.22
		*R*_2_	14.646	69.5				
		*R*_3_	15.297	72.5				
Plastic baby feeding bottle	0.1 μM, 25 μL	*R*_1_	23.351	110.8	96.3	13.338	13.84	7.70
		*R*_2_	19.747	93.7				
		*R*_3_	17.812	84.5				
Polythene food packaging	0.1 μM, 25 μL	*R*_1_	10.875	51.6	48.7	2.559	5.25	1.47
		*R*_2_	10.074	47.8				
		*R*_3_	9.849	46.7				
Mineral water bottle	0.1 μM, 25 μL	*R*_1_	12.718	60.3	54.4	5.301	9.72	3.06
		*R*_2_	11.185	53.0				
		*R*_3_	10.544	50.0				

a SD = standard deviation; RSD = relative standard deviation; SEM = standard error of mean.

## 4. Conclusions

A new NDNA Schiff base was easily synthesized, and its molecular structure was confirmed by singly-crystal X-ray diffraction studies to have the dimensions of *a* = 20.7011(17) Å, *b* = 6.2155(4) Å, *c* = 18.0683(13) Å, space group = *Pca*2 and *Z* = 4. The final *R*_2_ was 0.1369 (all data), and *R*_1_ was 0.0543 (*I* > 2\*s*(*I*)). We observed inter- and intra-molecular hydrogen bonding interactions which provide stability to the crystal structure. The N–H⋯O and O–H⋯N type intramolecular interactions form the six-membered ring motifs. The NDNA was used to fabricate a new electrochemical sensor (NDNA/nafion/GCE) for the detection of Sb^3+^ from simulated as well as real water samples in the presence of other interfering heavy metal cations by using the *I*–*V* method. This novel study gives a good proposal for the selective and very sensitive detection of Sb^3+^ analytes by using NDNA/nafion/GCE as an antimony probe with short response time and good reproducibility as well as repeatability. Hence, this technique launches a new approach that can be applied for the development of new electrochemical sensors for probing other heavy metal cations in environmental and healthcare fields.

## Conflicts of interest

The authors declare no conflict of interest.

## Supplementary Material

RA-008-C8RA01827H-s001

RA-008-C8RA01827H-s002
