# Supplementary material for: Fabrication of Sb3+ sensor based on 1,1′-(-(naphthalene-2,3-diylbis(azanylylidene))bis(methanylylidene))bis(naphthalen-2-ol)/nafion/glassy carbon electrode assembly by electrochemical approach
Source: RSC Adv. 2018 May 29;8(35):19754–64. doi: 10.1039/c8ra01827h (PMC9080665; doi:10.1039/c8ra01827h)
Supplement: RA-008-C8RA01827H-s001 [file RA-008-C8RA01827H-s001.pdf]

Electronic Supplementary Material (ESI) for RSC Advances.
This journal is © The Royal Society of Chemistry 2018

Electronic Supplemental Material (ESM)

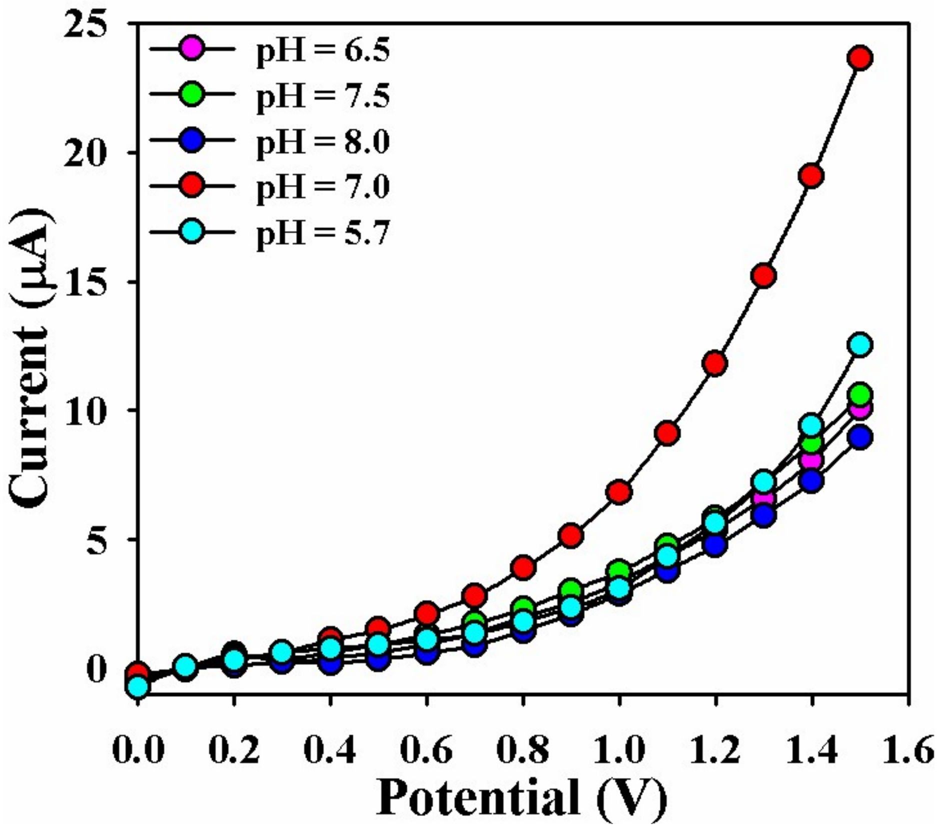

Fig. S1: Optimization of operational condition (Effect of pH).

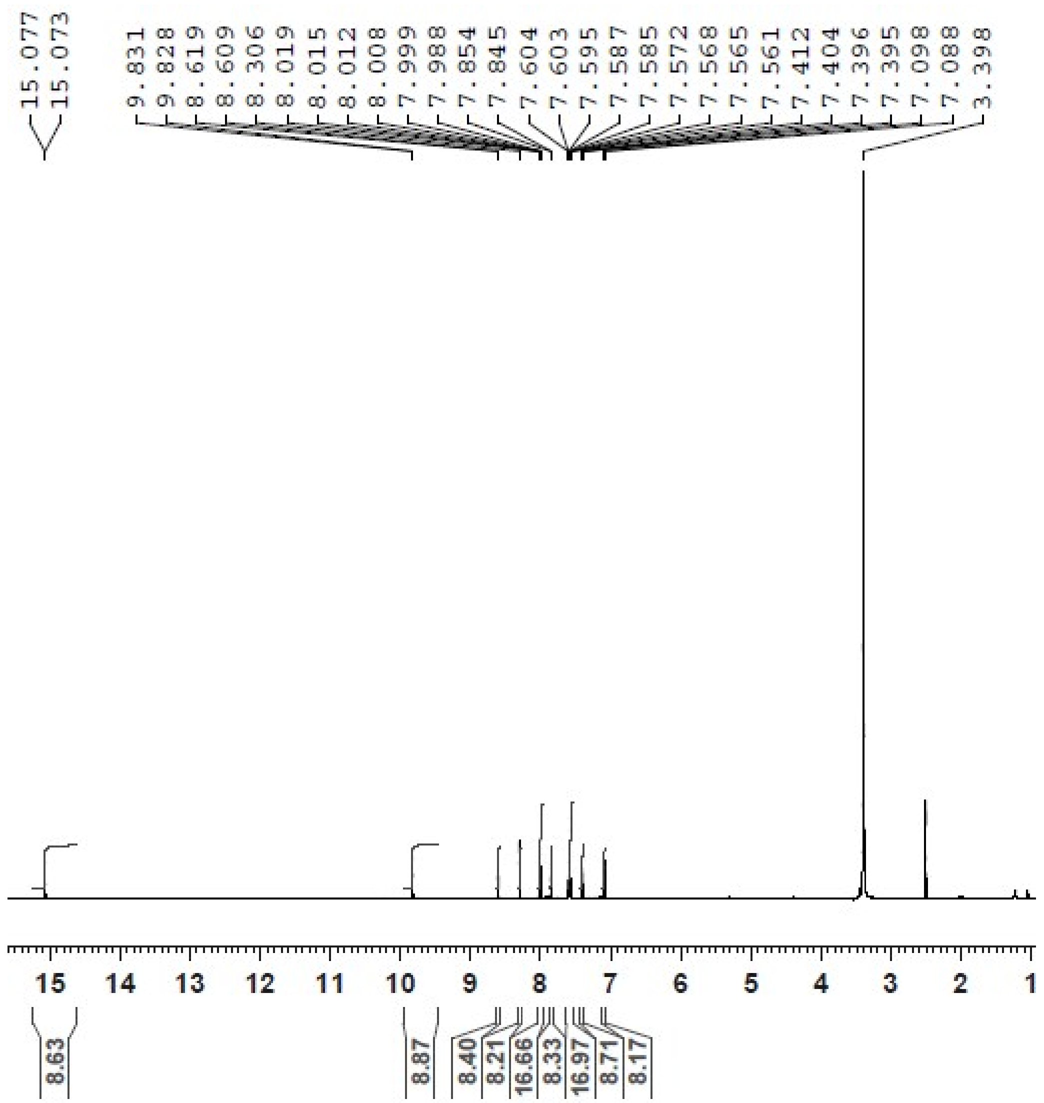

Fig. S2: HNMR of NDNA DMSO-d_6.

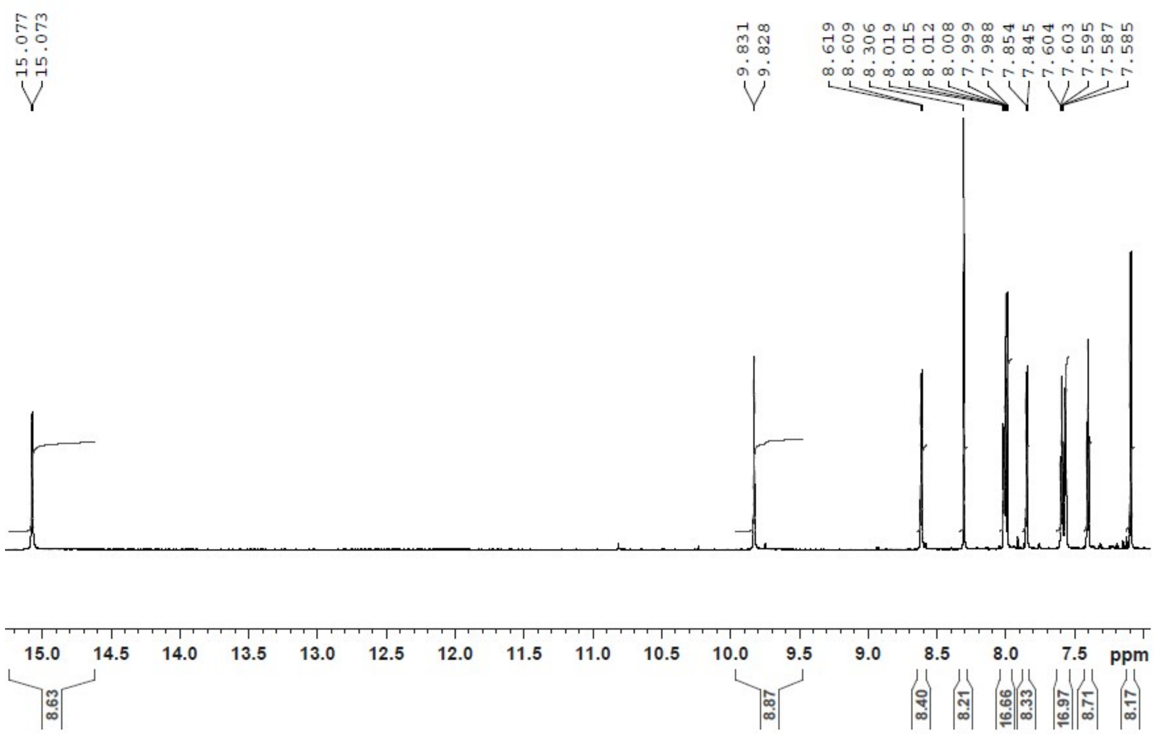

Fig. S3: ^{1}HNMR of NDNA in DMSO-d_6.

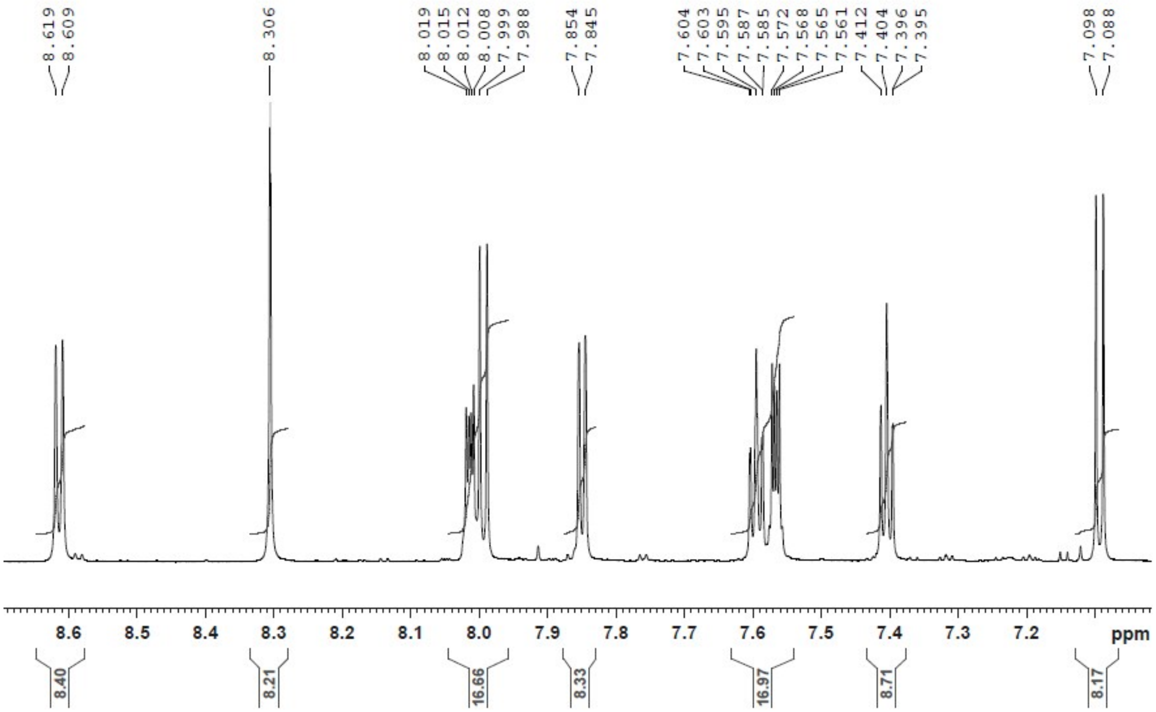

Fig. S4: ^{1}HNMR of NDNA in DMSO-d_6.

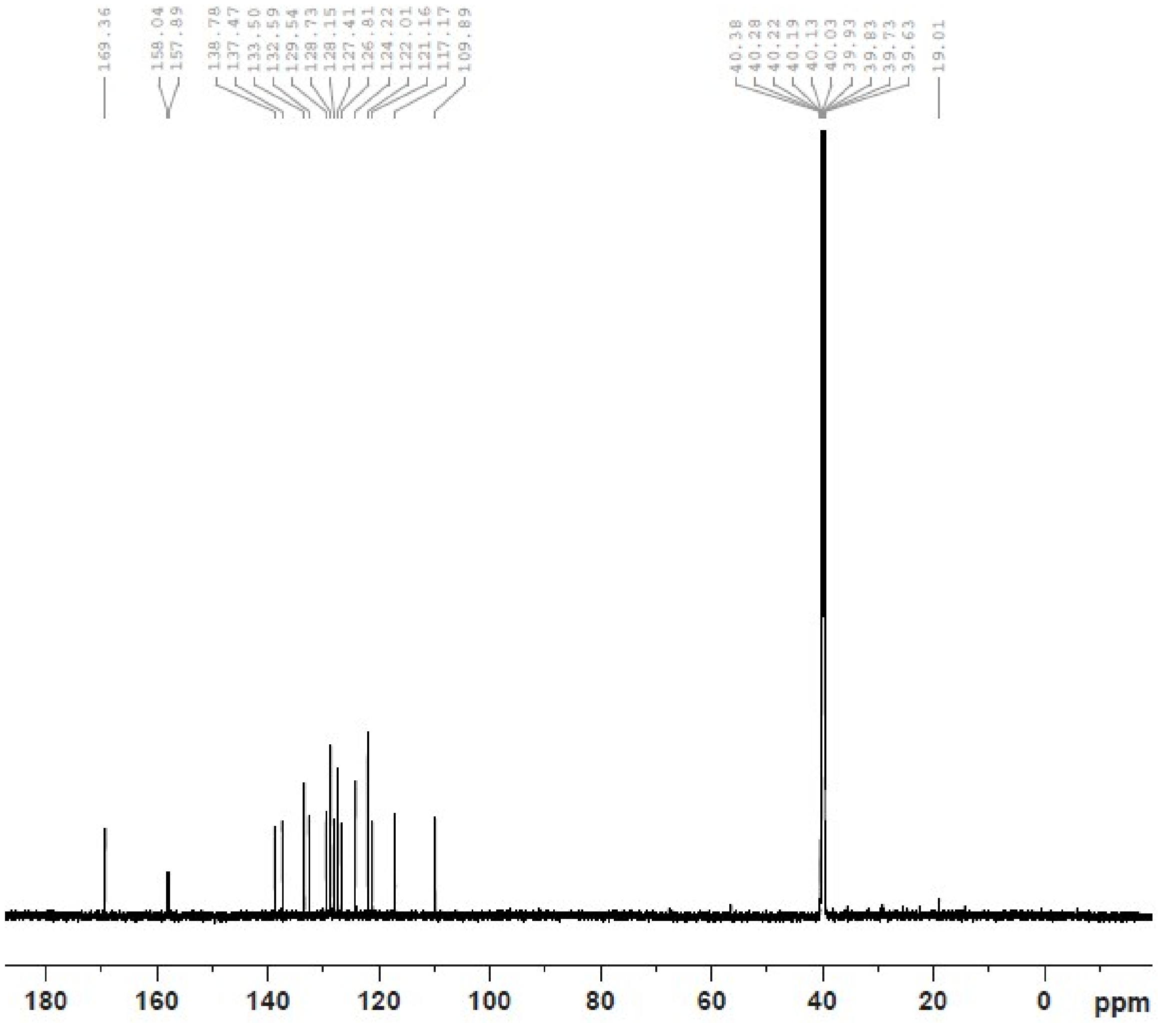

Fig. S5: ^{13}CHNMR of NDNA in DMSO-d_6.

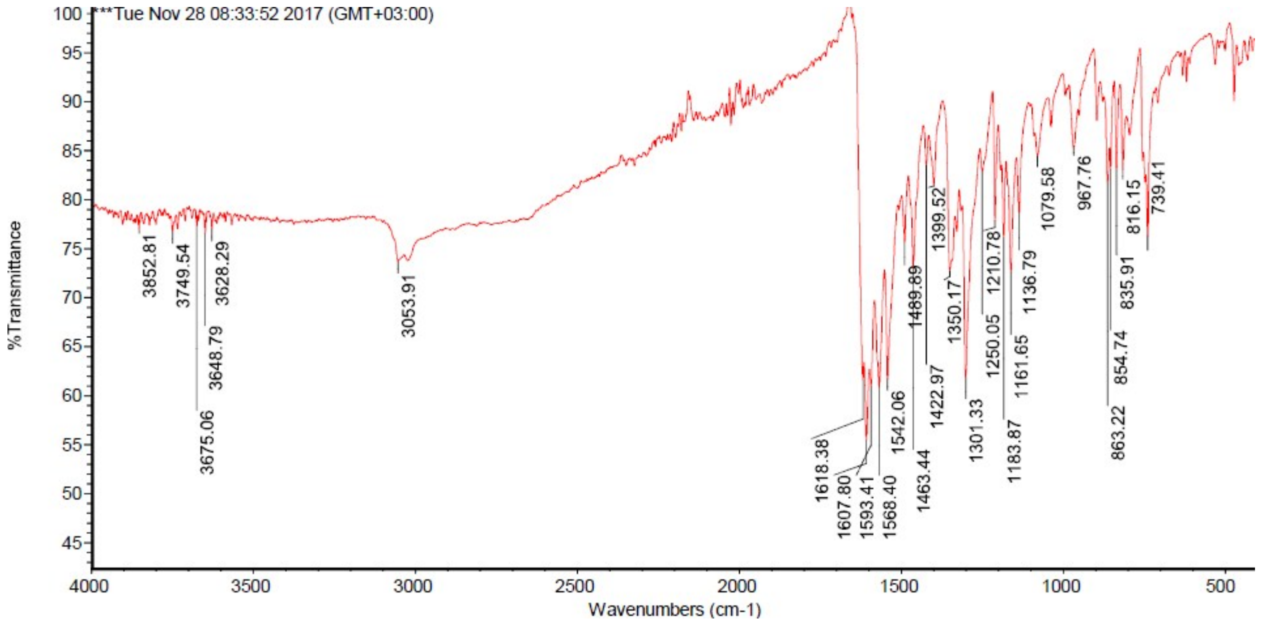

Fig. S6: ATR-FTIR of NDNA.

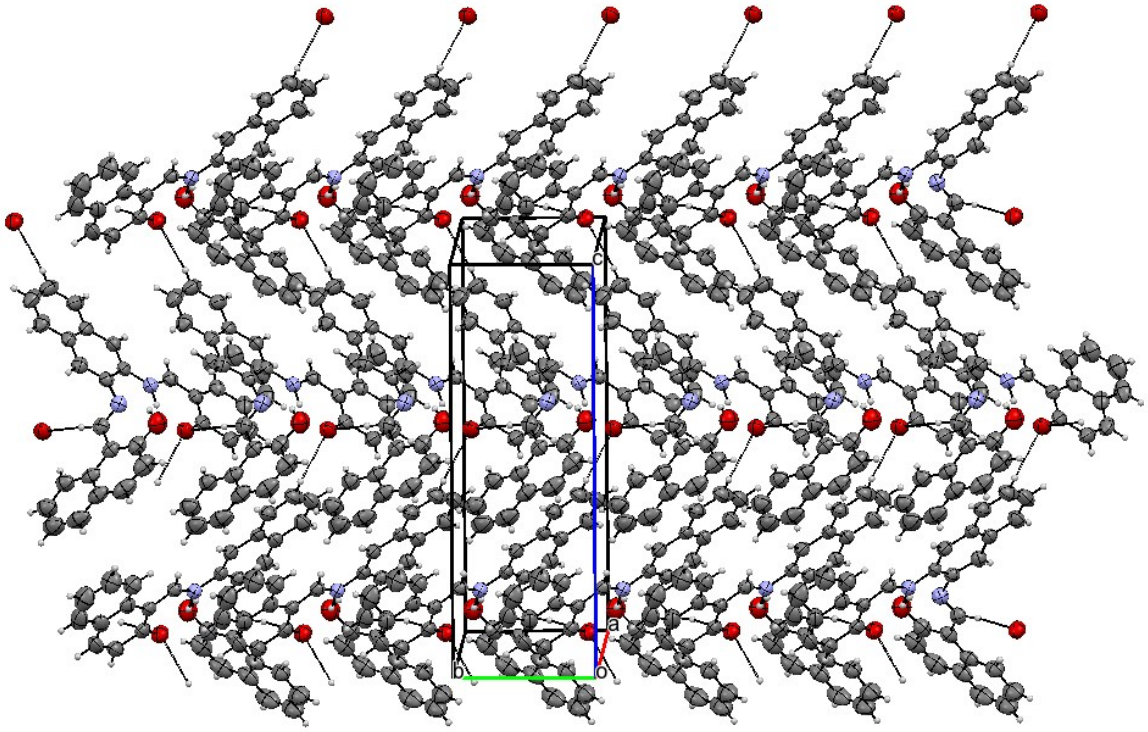

Fig. S7: A view of the formation of two-dimensional network through the intermolecular hydrogen bonding.

Table S1: Selected bond lengths for 17082.

Atom	Atom	Length/Å		Atom	Atom	Length/Å
C1	C2	1.360(5)		C16	C17	1.403(6)
C1	C10	1.434(5)		C16	C21	1.411(7)
C1	N2	1.424(5)		C17	C18	1.398(6)
C2	C3	1.418(5)		C18	C19	1.374(6)
C3	C4	1.413(5)		C19	C20	1.373(8)
C3	C8	1.422(6)		C20	C21	1.357(8)
C4	C5	1.357(6)		C22	C23	1.447(6)
C5	C6	1.403(7)		C22	N2	1.281(5)
C6	C7	1.357(6)		C23	C24	1.373(6)
C7	C8	1.406(6)		C23	C28	1.440(6)
C8	C9	1.412(5)		C24	C25	1.416(6)
C9	C10	1.366(5)		C24	O2	1.342(6)
C10	N1	1.397(5)		C25	C26	1.359(8)
C11	C12	1.385(5)		C26	C27	1.400(7)
C11	N1	1.330(5)		C27	C28	1.428(6)
C12	C13	1.437(6)		C27	C32	1.392(7)
C12	C17	1.461(5)		C28	C29	1.400(6)
C13	C14	1.433(6)		C29	C30	1.367(6)
C13	O1	1.271(5)		C30	C31	1.411(8)

C14	C15	1.341(7)	C31	C32	1.352(7)
C15	C16	1.423(7)			

Table S2:Selected bond angles for 17082.

Atom	Atom	Atom	Angle/°	Atom	Atom	Atom	Angle/°
C2	C1	C10	119.8(4)	C21	C16	C15	121.3(5)
C2	C1	N2	122.8(4)	C16	C17	C12	119.0(4)
N2	C1	C10	117.4(3)	C18	C17	C12	123.1(4)
C1	C2	C3	122.0(4)	C18	C17	C16	117.8(4)
C2	C3	C8	118.1(4)	C19	C18	C17	121.0(5)
C4	C3	C2	123.2(4)	C20	C19	C18	121.1(5)
C4	C3	C8	118.7(4)	C21	C20	C19	119.4(5)
C5	C4	C3	120.9(4)	C20	C21	C16	121.2(5)
C4	C5	C6	120.2(5)	N2	C22	C23	122.3(4)
C7	C6	C5	120.5(5)	C24	C23	C22	119.7(4)
C6	C7	C8	121.1(5)	C24	C23	C28	120.2(4)
C7	C8	C3	118.5(4)	C28	C23	C22	119.8(4)
C7	C8	C9	122.3(4)	C23	C24	C25	120.3(5)
C9	C8	C3	119.2(4)	O2	C24	C23	123.3(4)
C10	C9	C8	121.6(4)	O2	C24	C25	116.4(5)
C9	C10	C1	119.4(3)	C26	C25	C24	119.5(5)

C9	C10	N1	124.5(4)	C25	C26	C27	122.9(5)
N1	C10	C1	116.1(3)	C26	C27	C28	118.1(5)
N1	C11	C12	123.4(4)	C32	C27	C26	122.6(5)
C11	C12	C13	119.4(4)	C32	C27	C28	119.3(5)
C11	C12	C17	121.1(4)	C27	C28	C23	118.7(4)
C13	C12	C17	119.4(3)	C29	C28	C23	123.8(4)
C14	C13	C12	118.4(4)	C29	C28	C27	117.5(5)
O1	C13	C12	122.0(4)	C30	C29	C28	121.6(5)
O1	C13	C14	119.6(4)	C29	C30	C31	120.5(6)
C15	C14	C13	121.0(4)	C32	C31	C30	118.8(5)
C14	C15	C16	122.8(4)	C31	C32	C27	122.4(5)
C17	C16	C15	119.3(4)	C11	N1	C10	125.8(4)
C17	C16	C21	119.4(5)	C22	N2	C1	119.3(3)
